# Birt–Hogg–Dubé syndrome with rare unclassified renal cell carcinoma: A case report

**DOI:** 10.1097/MD.0000000000028380

**Published:** 2021-12-23

**Authors:** Shangqing Ren, Cheng Luo, Yaoqian Wang, Yi Wei, Yong Ou, Jiazheng Yuan, Xinglan Li, Junyao Wang, Qian Lv, Bo Yang, Shida Fan, Fang Zhou, Zhengjun Chen, Yu Nie, Dong Wang

**Affiliations:** a Department of Robotic Minimally Invasive Surgery Center, Sichuan Academy of Medical Sciences & Sichuan Provincial People's Hospital, Chengdu, Sichuan, China; b Department of Pathology, Sichuan Provincial People's Hospital, University of Electronic Science and Technology of China, Chengdu, China; c Chinese Academy of Sciences Sichuan Translational Medicine Research Hospital, Chengdu, China.; d Department of Pediatric Surgery, Sichuan Academy of Medical Sciences and Sichuan Provincial People's Hospital, Chengdu, Sichuan, China.

**Keywords:** Birt-Hogg-Dubé syndrome, case report, renal cell carcinoma, unclassified renal cell carcinoma

## Abstract

**Introduction:**

Birt-Hogg-Dubé syndrome (BHDS) is a rare genetic disease. Renal cell carcinoma is the most serious complication of BHDS. The histological types of BHDS-related renal cell carcinoma are mostly mixed chromophobe/eosinophil and chromophobe cell types. BHDS with unclassified renal cell carcinoma is extremely rare.

**Patient concerns:**

A 37-year-old man was admitted to the hospital because of lumbago and hematuria.

**Diagnosis:**

Combined with abdominal enhanced CT and pulmonary CT, BHDS complicated with renal cell carcinoma was diagnosed, and right partial nephrectomy was performed. The postoperative pathological diagnosis was unclassified renal cell carcinoma. Gene detection revealed the FLCN frameshift mutation.

**Outcomes:**

No signs of recurrence were observed after regular follow-up.

**Conclusion:**

The pathogenesis of BHDS has not been fully elucidated, and the pathological type of BHDS with unclassified renal cell carcinoma is extremely rare. Through case presentation and review of related literature, this paper summarizes the diagnosis and treatment of BHDS complicated with unclassified renal cell carcinoma.

## 1 Introduction

Birt-Hogg-Dubé syndrome (BHDS) is an autosomal dominant hereditary disease associated with folliculin (FLCN) gene mutation on chromosome 17. It was first reported and named by Birt et al in 1993, but its pathogenesis has not been fully elucidated. The main clinical manifestations of the disease are benign tumors of the skin, cystic changes in the lungs, and various types of renal tumors. Among them, renal cell carcinoma (RCC) is the most serious complication of BHDS, which often occurs in multifocal or bilateral cases. Most of them have slow growth, low invasiveness, and occasional metastasis.^[1]^ There are various tissue types of BHDS-related RCC, and multiple tumors in the same patient may present different pathological types, with more mixed chromophobe/oncocytomas and chromophobe cell types, and fewer clear cell and papillary RCC types.^[2]^ As the World Health Organization (WHO) updated the pathological classification of renal tumors in 2016, there have been no cases of BHDS with unclassified RCC. Surgery is the main treatment for BHD complicated with RCC, and it has also been reported that the mechanistic target of rapamycin (mTOR) inhibitor everolimus can be used in the treatment of metastatic RCC,^[3,4]^ but its efficacy evaluation requires further research.

## 2 Case presentation

**Figure 1:**
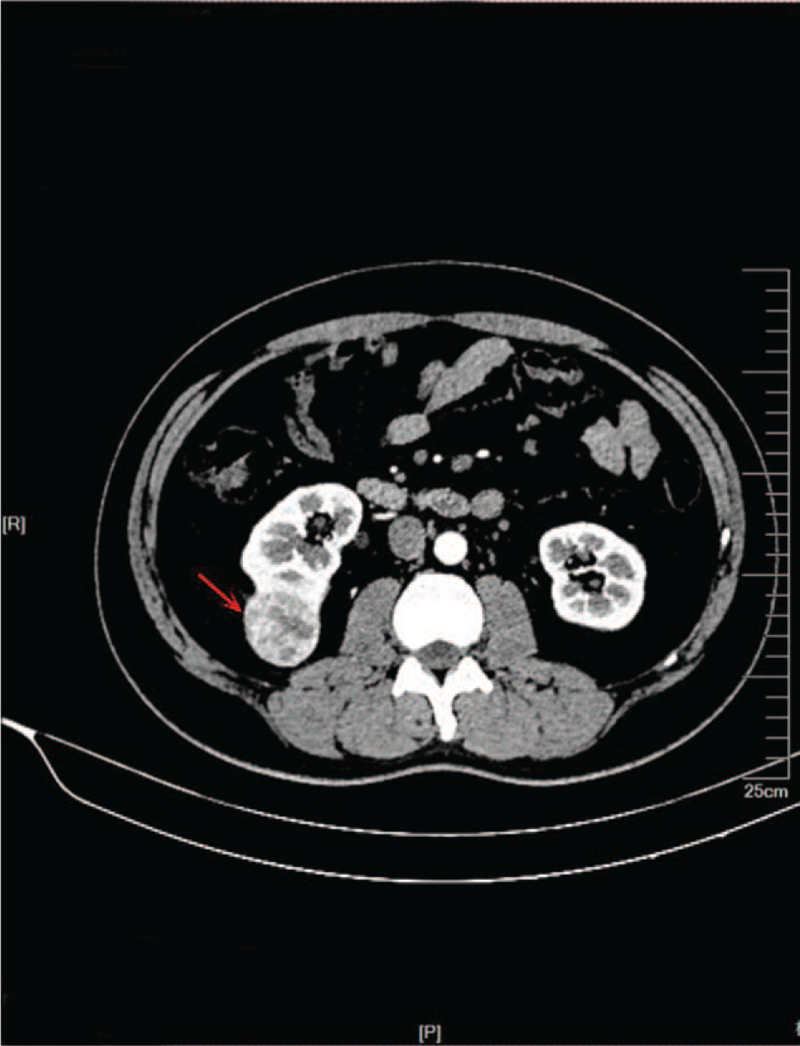
CT abdomen: A quasi-circular slightly high-density mass in the lower part of the right kidney, with calcification in the lower part of the right kidney, protruding the outline of the kidney and showing obvious inhomogeneous enhancement in the arterial phase.

**Figure 2:**
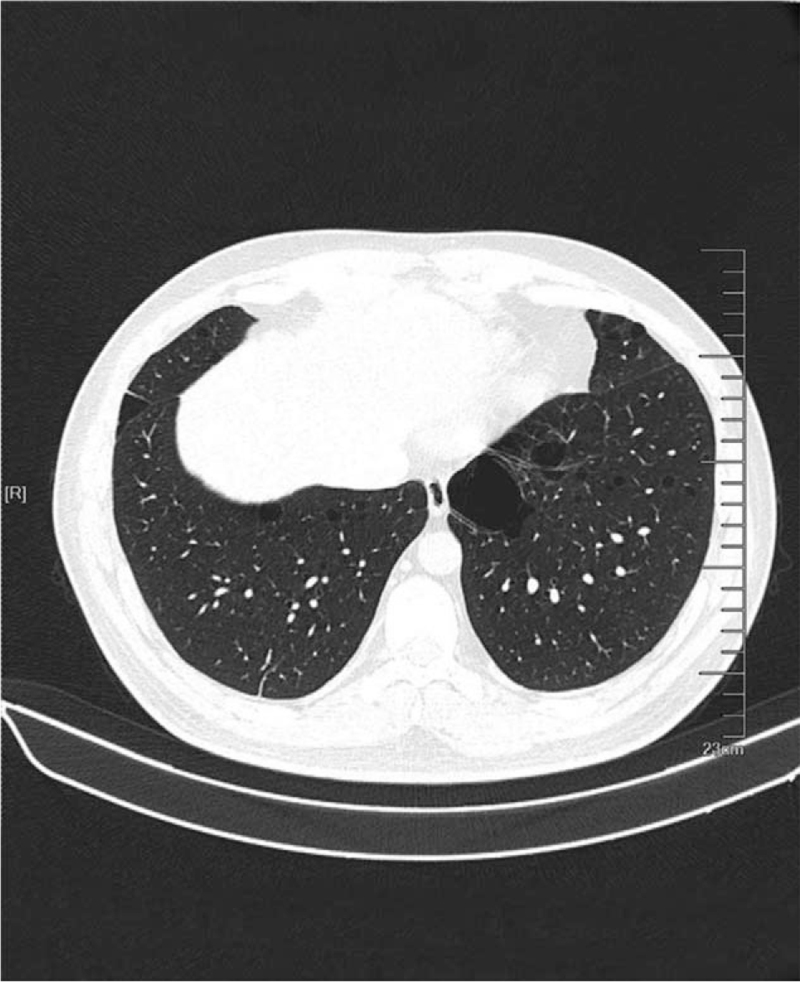
Plain Chest CT: multiple cystic low-density shadows in both lungs were irregular in size.

**Figure 3:**
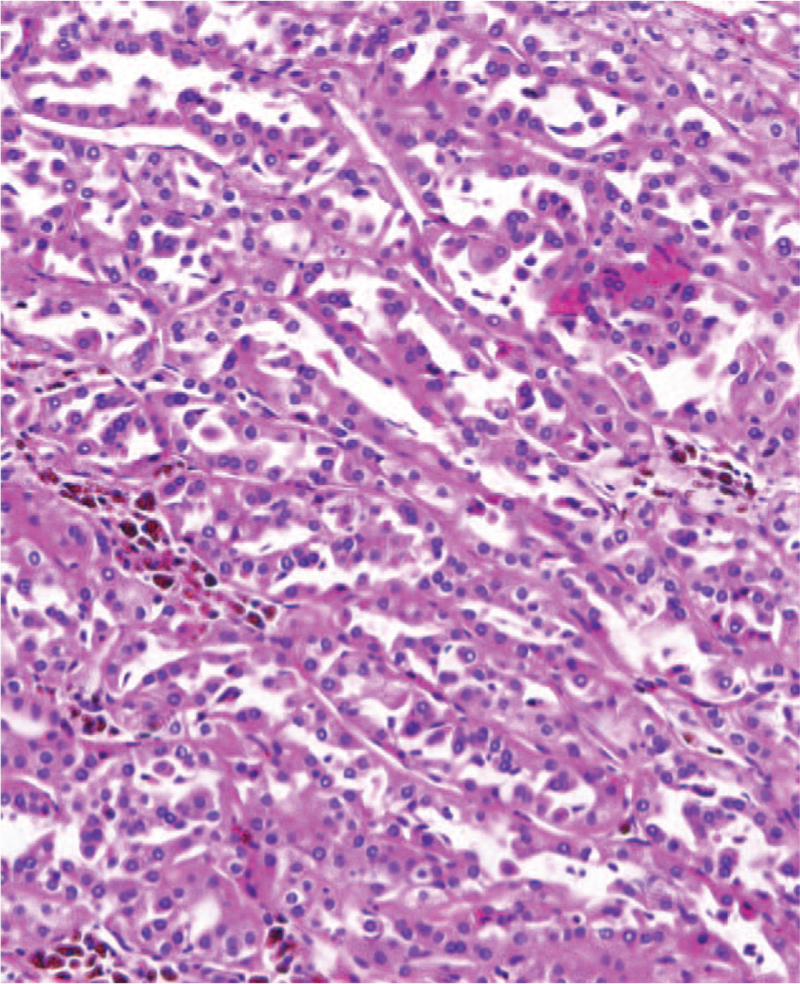
Histopathology: HE(1-10x20) microscopy showed a diagnosis consistent with unclassified renal cell carcinoma by WHO in 2016.

**Figure 4:**
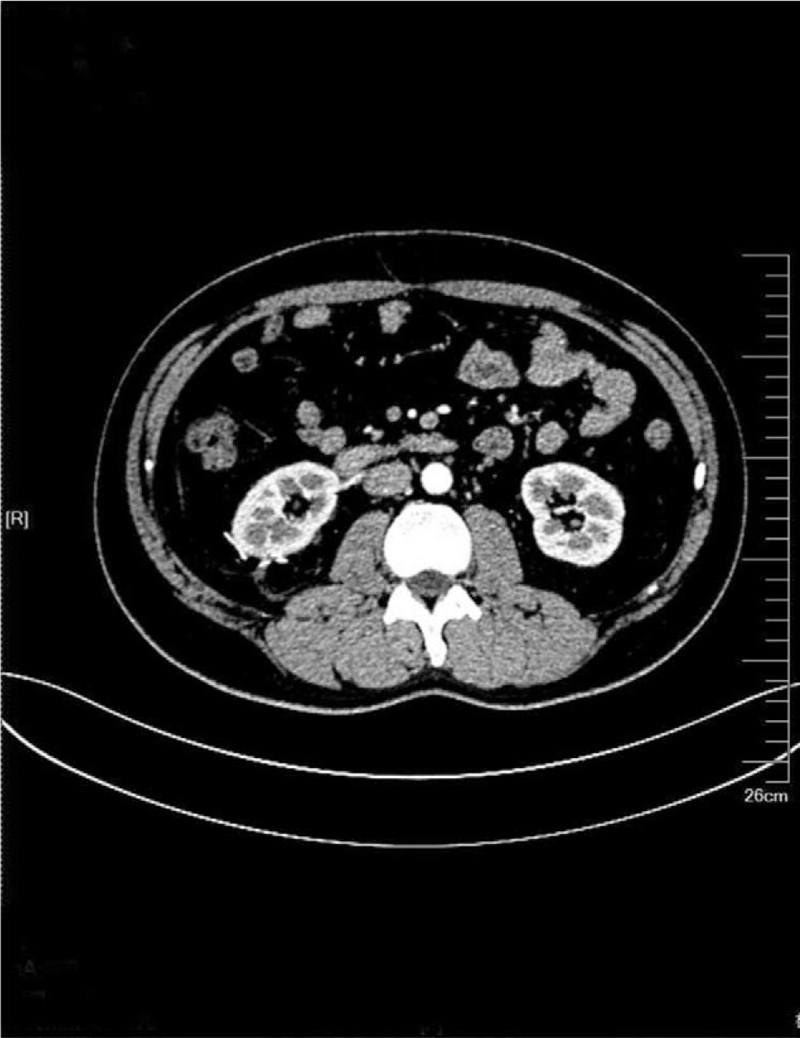
CT abdomen: no obvious signs of recurrence.

A male, 37 years old, was admitted to the hospital in September 2020 because of bilateral lumbago for 6 days and aggravation with hematuresis for 2 days. He had a previous medical history of spontaneous pneumothorax, and his father had a history of spontaneous pneumothorax. Color Doppler ultrasound of the urinary system showed a slightly hyperechoic mass of approximately 53 × 37 × 31 mm in size was detected in the middle part of the right kidney, protruding to the outside of the capsule, and the internal echo was non-homogeneous and the blood flow signal could be seen. Contrast-enhanced computed tomography (CT) of the whole abdomen (Fig. 1) suggested that there was a quasi-circular, slightly high-density mass in the lower part of the right kidney, with calcification in the lower part of the right kidney, with a maximum diameter of approximately 39 mm, protruding the outline of the kidney and showing obvious inhomogeneous enhancement in the arterial phase. Plain chest CT (Fig. 2) showed that multiple cystic low-density shadows in both lungs were irregular in size, mainly in the lower part, subpleural, and adjacent to the interlobar fissure, with a maximum diameter of approximately 13.6 mm. Robot-assisted laparoscopic partial nephrectomy was performed under general anesthesia on September 15, 2020. During the operation, a 40 × 40 × 30 mm mass was observed on the dorsal side of the middle and inferior pole of the right kidney, part of which was endogenetic; the boundary was unclear, and fish-like new organisms and scattered necrotic hemorrhagic foci could be seen in the specimen profile. Postoperative pathological diagnosis (Fig. 3) revealed that the maximum diameter of the tumor was approximately 55 mm, and there was no tumor infiltration at the cutting edge of the specimen. Combined with morphology, immunophenotype, and related gene detection, it was considered as unclassified RCC. Among them, the postoperative gene detection showed that the gene FLCN frameshift mutation, the mutation result c. 946–947del (p.s316fs), had an abundance of 54.49%. Regular follow-up was performed every 3 months after discharge, and the recovery was very good, and abdominal CT (Fig. 4) showed no obvious signs of recurrence.

## 3 Discussion

BHDS is an autosomal dominant genetic disease characterized by benign skin tumors, cystic changes in the lungs, and various types of renal tumors. Pulmonary cystic change, which is observed in 90% of patients, is the most common manifestation of BHDS. It is usually irregular, mostly at the base of the lung, rarely at the tip of the lung, and varies in number and size.^[5]^ Kumasaka et al^[6]^ believe that the loss of FLCN leads to the weakening of the alveolar wall, which is easily destroyed by mechanical pressure during respiration, which leads to cystic changes. This paper reports a history of pneumothorax, combined with his father's history of spontaneous pneumothorax, consistent with the characteristics of the genetic diagnosis.

RCC is the most serious complication of BHDS, usually bilateral or multiple, with slow growth, low invasiveness, and occasional metastasis. The incidence in BHDS patients is approximately 30%, and the risk of kidney cancer is approximately seven times higher than that in the general population.^[7]^ BHDS-related renal tumors are histologically diverse. A study analyzed the histology of 130 renal tumors removed from 30 BHDS patients and found that 50% were mixed chromophobe/oncocytomas, 34% were chromophobe cells, 9% were clear cells, 5% were oncocytomas, and 2% were papilloma.^[8]^ In addition, many studies have also reported that the pathological type of BHDS complicated with RCC is mixed chromophobe/oncocytomas, and chromophobe cells are common. Over the past few decades, due to the in-depth study of genetics and morphology and more accurate detection instruments, the classification of RCC has experienced continuous improvement. The WHO first introduced the concept of unclassified RCC in 1997, and RCC that did not belong to the four common types known at that time were classified into this category. In 2004, the WHO further specified the characteristics of this type of RCC: a mixture of known types of RCC, pure sarcomatoid morphology without recognizable epithelial components, mucin production, a rare mixture of epithelial and matrix components, and unrecognizable cell types. In 2016, the WHO released the latest classification of RCC, including 6 new subtypes of RCC and four provisional subtypes of RCC, further narrowing the scope of unclassified RCC.^[9–11]^ This is the first case of BHDS complicated by unclassified RCC since the latest WHO classification in 2016.

FLCN gene detection is the most significant diagnostic method for BHDS. This patient was also definitively diagnosed with the results of a gene test for FLCN abnormalities. Fibrofollicular tumor or trichodiscoma diagnosed by skin pathological biopsy is another major criterion for the diagnosis of BHDS. The diagnosis of BHDS-related pulmonary cysts usually depends on CT, and high-resolution CT is recommended for differentiation from other cystic lung diseases.^[5]^ It is recommended that the monitoring age of BHDS-related renal cell carcinoma should start from 20 years of age, and CT and MRI are recommended. However, because of the long surveillance period of the disease, repeated CT may produce an unacceptably high cumulative radiation dose, so MRI seems to be the best surveillance method.^[12,13]^ In addition, clinical symptoms and family genetic history are still very important in the diagnosis of BHDS.

At present, there is no cure for BHDS, and the treatment of pneumothorax is the same as that in ordinary pneumothorax patients; for patients with recurrent pneumothorax, pleural adhesion can be considered to reduce the risk of recurrent pneumothorax. The degree of malignancy and invasiveness of most BHDS-related renal tumors are relatively low, but the recurrence rate is high and often multiplies. For long-term preservation of renal function, surgical resection should be conservative and cautious. Follow-up observation or percutaneous thermal ablation is usually recommended for renal tumors measuring < 3 cm. Nephron-sparing partial nephrectomy is preferred for renal tumors measuring > 3 cm.^[5]^

The pathogenic genes FLCN and its 2 proteins FNIP1 (Folliculin-interactingprotein1) and FNIP2 (Folliculin-interacting protein2) play a synergistic role in metabolic pathways, including AMPK-mediated energy sensing, mTORC1-dependent cell proliferation, and Ppargc1a-driven mitochondrial oxidation, and disorders in these metabolic pathways trigger abnormal renal cell proliferation and renal tumorigenesis.^[14]^ Further studies on FLCN function, such as targeted therapy with mTOR inhibitors for BHDS-related renal cell carcinoma have been reported.^[15]^ The National Institutes of Health recently announced a new clinical trial on the use of the mTOR inhibitor everolimus in the treatment of patients with RCC. Everolimus has been approved for the treatment of sporadic metastatic RCC, but it has not been specifically tested in BHDS-related and sporadic chromophobe cell RCC. Cases of BHDS complicated with unclassified RCC are extremely rare and require multidisciplinary cooperation to develop individualized treatment. At present, surgical resection is still the main method, and mTOR inhibitors can be used if necessary, but the clinical efficacy remains to be reported and prospectively studied in more cases of BHDS with unclassified RCC.

## 4 Conclusion

BHDS is a rare genetic disease. FLCN is the only related gene known to date, and its pathogenesis has not been fully elucidated. RCC is bilateral or multiple, the pathological type is mixed chromophobe/oncocytomas, chromophobe cells are common, and the pathological type of undifferentiated RCC BHDS is extremely rare and requires multidisciplinary cooperation to develop individualized treatment, still dominated by surgical treatment. Clinically, early diagnosis, early treatment, and long-term follow-up are the primary methods. The choice of examination and follow-up methods can be individualized. In terms of lifestyle, patients with pneumothorax should avoid diving, flying, and strenuous exercise as far as possible.

## Acknowledgments

The authors would like to express my gratitude to Dr Wang Dong, who provided me with valuable guidance for this article.

## Author contributions

SQR and CL were responsible for writing and editing the article; YQW, YW, YO, and JZY were responsible for reviewing the literature; XLL,YJW,QL,BY,SDF,FZ,ZJC, and YN were responsible for collecting the information of the current case. DW was responsible for revising the manuscript for important intellectual content. All authors have read and approved the manuscript.

**Data curation:** Shangqing Ren, Cheng Luo, Yaoqian Wang, Yi Wei, Yong Ou, Jiazheng Yuan, Xinglan Li, Junyao Wang, Qian Lv, Bo Yang, Shida Fan, Fang Zhou, Zhengjun Chen, Yu Nie.

**Investigation:** Shangqing Ren, Cheng Luo, Yaoqian Wang, Yi Wei, Yong Ou, Jiazheng Yuan.

**Project administration:** Dong Wang.

**Writing – original draft:** Cheng Luo.

**Writing – review & editing:** Shangqing Ren, Cheng Luo, Dong Wang.
